# Prevention of Chronic Kidney Disease and Subsequent Effect on Mortality: A Systematic Review and Meta-Analysis

**DOI:** 10.1371/journal.pone.0071784

**Published:** 2013-08-29

**Authors:** Usman A. Khan, Amit X. Garg, Chirag R. Parikh, Steven G. Coca

**Affiliations:** 1 Section of Nephrology, Yale University School of Medicine, Veterans Affairs Medical Center, and the Program of Applied Translational Research, New Haven, Connecticut, United States of America; 2 Division of Nephrology, Department of Medicine, Western University, London, Ontario, Canada; University of British Columbia, Canada

## Abstract

**Objectives:**

To perform a systematic review of randomized controlled trials to determine whether prevention or slowing of progression of chronic kidney disease would translate into improved mortality, and if so, the attributable risk due to CKD itself on mortality.

**Background:**

CKD is associated with increased mortality. This association is largely based on evidence from the observational studies and evidence from randomized controlled trials is lacking.

**Methods:**

We searched Ovid, Medline and Embase for RCTs in which an intervention was given to prevent or slow the progression of CKD and mortality was reported as primary, secondary or adverse outcomes were eligible and selected. For the first phase, pooled relative risks for renal endpoints were assessed. For the second phase, we assessed the effect on mortality in trials of interventions that definitively reduced CKD endpoints.

**Results:**

Among 52 studies selected in first phase, only renin-angiotensin-aldosterone-system blockade vs. placebo (n = 18 trials, 32,557 participants) met the efficacy criteria for further analysis in the second phase by reducing renal endpoints 15 to 27% compared to placebo. There was no difference in all-cause mortality (RR 0.99, 95% CI 0.92 to 1.08) or CV death (RR 0.97, 95% CI 0.78 to 1.21) between the treatment and control groups in these trials. There was sufficient statistical power to detect a 9% relative risk reduction in all-cause mortality and a 14% relative risk reduction in cardiovascular mortality.

**Conclusions:**

Firm evidence is lacking that prevention of CKD translates into reductions in mortality. Larger trials with longer follow-up time are needed to determine the benefit of CKD prevention on survival.

## Introduction

Chronic kidney disease (CKD) represents an increasing burden on health care systems worldwide. The prevalence of CKD has increased over the past several years. It is currently estimated that 17% of people in the United States have CKD, and worldwide the prevalence is 23–36% in people aged ≥64 [1], [2]. Over the past several years, it has been generally accepted in the medical literature and community that CKD is independently associated with premature mortality [3]–[5].

In order to confirm that a nontraditional factor, such as CKD, is a causal risk factor for mortality, the following conditions should be met: (i) biological plausibility as to why the factor may promote premature mortality; (ii) demonstration that the mortality risk increases with severity of CKD; (iii) demonstration of an association between the CKD and mortality in observational studies; and (iv) demonstration in placebo-controlled clinical trials that treatment of CKD decreases mortality. There is an abundance of evidence for first three conditions, however, the veracity of the last condition is largely unproven.[6]–[10] Randomized controlled trials eliminate the possibility that other conditions such as diabetes and hypertension, which cause CKD, confound the observed association between CKD and mortality. This is with the recognition though that an intervention’s effects may be complex, impacting an outcome such as mortality through many potential pathways one of which may be CKD prevention. A sufficient amount of time would also need to elapse since an intermediate endpoint such as CKD was prevented, before one would expect to see a reduction in subsequent attributable deaths.

Furthermore, even evidence from recent observational studies also questions the causal relationship between decreased glomerular filtration rate (GFR) and mortality. Garg et al. demonstrated the risk for cardiovascular events and death in people who donate a kidney were no higher in the first decade after transplantation than in matched non-donors [11]. Wald et al. used a large Ontario database to perform a propensity score matched cohort analysis and found that survivors of acute kidney injury that required dialysis had a significantly elevated risk for development of end stage renal disease (adjusted hazard ratio 3.2) but all-cause mortality rates were not elevated (adjusted hazard ratio 0.95) [12].

With this background, we performed a systematic review and meta-analysis of randomized controlled trials (RCTs) to determine whether interventions that are efficacious for reducing the incidence or progression of CKD result in a commensurate reduction in mortality (cardiovascular or all-cause).

## Methods

We used a standardized protocol to search the published literature and identify trials for our analysis.

### Literature Sources and Search Terms

We performed an exhaustive search and evaluation of peer-reviewed research published between 1948 and July 2011, including Ovid, MEDLINE and Scopus (EMBASE). We used many search terms and filters that include “exp renal insufficiency, chronic”, “hypertension, renal”, “proteinuria”, “diabetic nephropathies”, “disease progression”, “survival analysis”, “treatment outcomes”, “mortality.mp.” and “randomized controlled trials”. The search was limited to randomized controlled trials that studied human subjects without language restriction. All efforts were made to obtain the English translation of the trials published in non-English languages.

### Study Selection and Data Collection Process

We included trials with both CKD and non-CKD participants. We included trials that reported CKD outcomes along with mortality as primary, secondary or adverse outcomes. All included trials had a mean/median follow up time of at least 1 year and a sample size of at least 100 total participants. Two authors independently reviewed the references and resolved disagreements by discussion. The authors analyzed the quality of reporting by using the Cochrane Collaboration’s tool for assessing risk of bias: randomization method, allocation concealment, blinding of participants, staff and assessors, selective reporting (for renal endpoints), and description of withdrawals. [13] After careful assessment, we included a total of 52 trials for data abstraction.

### Data Abstraction

We used a standardized data abstraction form for description of the trial, such as the title of the study, the authors, year of publication, country, trial design, number of participants and their baseline characteristics. We also abstracted the type of intervention and the incidence of primary and secondary outcomes in the control and treatment groups.

### Outcome Measures

The renal endpoints included the following: doubling of serum creatinine (sCr), End Stage Renal Disease (ESRD) defined as initiation of dialysis or renal transplant, composite of doubling of serum creatinine or ESRD, albuminuria/proteinuria (incidence, progression, regression). The non-renal endpoints were cardiovascular (CV) death and all-cause mortality.

### Statistical Analysis

Trials were grouped by type of intervention and then were analyzed in two phases. In the first phase of the analysis, pooled relative risks (RR) for each of the renal endpoints for each treatment vs. control were computed with Mantel-Haenszel statistics. Interstudy heterogeneity was calculated using the Chi^2^ method and the I^2^ statistic. *A priori*, we decided that if the pooled upper bound of the 95% confidence interval (CI) was <1 and at least 3 individual studies demonstrated efficacy for the renal endpoints as evidenced by an upper bound of the 95% CI <1, then the intervention was deemed “efficacious for prevention of incident or progressive CKD” and advanced to the second phase. We combined the trials evaluating either angiotensin converting enzyme inhibitors (ACEi) or angiotensin receptor blockers (ARBs) as one intervention called “RAAS blockade”. In the second phase, the association between the renoprotective intervention and all-cause and cardiovascular mortality was assessed. The results were considered significant with 2-sided α error <0.05. All the results are reported with 95% CIs. Statistical calculations and graphs were made using the Review Manager (RevMan), Version 5.1. Copenhagen: The Nordic Cochrane Center, The Cochrane Collaboration, 2011.

In addition, for the second phase, we performed sensitivity analyses that examined the pooled relative risk of CV death and all-cause mortality by pooling only the trials within the type of intervention that demonstrated efficacy for the treatment for the CKD outcome. We also explored the diversity in study results and possible association of certain covariates to hard outcomes by performing sub-group analyses and by using meta-regression. For each meta-regression and subgroup analysis, only studies for which the factor of interest was available were included in the analysis. The statistical significance was determined by the proportion of variability explained by each study level characteristic and from the size of residual variance [14]. Best-fit lines in meta-regression graphs were estimated by generalized estimating equations using estimates from meta-regression models as the input values and were weighted by the variance of each estimate [15]. Meta-regression analyses were conducted in SAS 9.3 and R 2.15.0.

## Results

**Figure 1:**
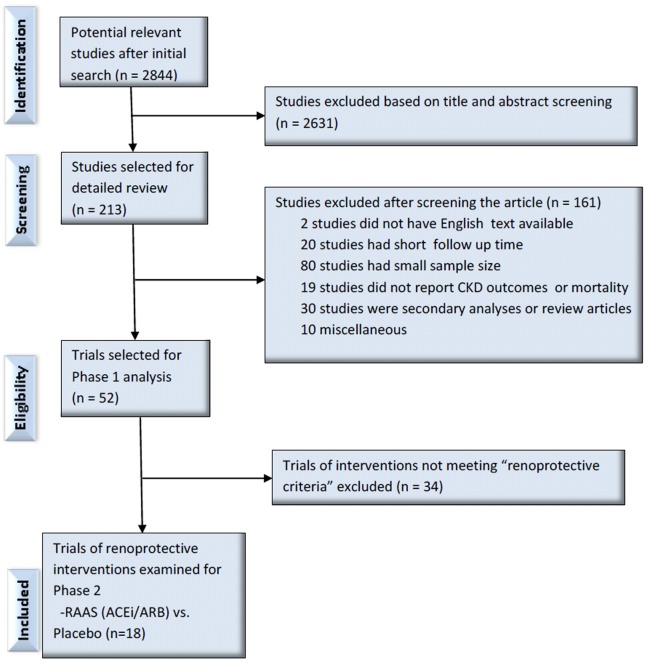
Flow Diagram of Study Selection Process.

Our search identified 2844 citations for the first phase. Based on title and abstract review, we excluded 2631 citations. After a detailed review of 213 citations we further excluded 161 for various reasons. Among those, 20 trials had follow up time less than a year, 80 trials had small sample size, 19 trials did not report CKD outcomes or mortality, 30 citations were review articles or secondary analyses and 10 were excluded based on miscellaneous reasons (Figure 1). We also were unable to obtain full text in English and excluded those 2 trials. After a careful and thorough review, we included 52 trials in phase 1 [16]–[70]. The characteristics of these trials are listed in Table 1.

**Table 1:** Baseline Study Characteristics of All Studies in Phase 1.

STUDY	Sample	Follow up	Age (y)	Male	Clinical	Baseline Renal Function Indicators				Co-Morbidities (%)			Country
	Size	in years (mean/median)	mean	(%)	Setting	sCr eGFR/CrCl MicroAlb MacroAlb mg/dl ml/min/1.73 m^2^ (%) (%)				DM HTN CVD			
UKPDS34 1998	753	10.7	53	46	T2DM	0.9 (0.7–1.1)	–	0	2.2	100			UK
UKPDS33 1998	3867	10	53.3	61	T2DM	0.9 (0.7–1.1)	–	0	1.9	100			UK
Rachmani R et al 2004	141	7.7	57.1	49	T2DM, HTN	–	106±9.5	100	0	100	100	0	Israel
DCCT 1993	1441	6.5	27	53	IDDM	–	128±30	0	0	100			USA, Canada
Kumamoto 1995	110	6	49	44.5	T2DM	–	–	46	0	100	0	0	Japan
VADT 2009	1791	5.6	60.4	97	T2DM, HTN	1.0±0.2	–	–	–	100	72	40	USA
4S	3842	5.5	60	69	CHD, CKD, DM	1.1±0.2	66.5±8.4	–	–	3.5	31	100	Europe, USA
AFCAPS-TexCAPS 2010	4994	5.3	60	81.7	HLP	1.1±0.2	71±12	–	–	2	28.5	0	USA
ADVANCE 2008	11140	5	66	57.5	T2DM, CVD, HTN	0.9±0.3	–	26.8	3.6	100	68.5	32	Multicontinental
Mauer M, et al 2009	256	5	29.9	46	T1DM	–	129±20	0	0	100	0		USA, Canada
SHARP 2011	9270	4.9	62	63	CKD	–	26.6±13	38	42	23	100	15	Multicontinental
ALLHAT_BP 2005	33357	4.8	67	52.3	HTN, CVD, T2DM, CKD	–	76±10	–	–	39	100	26	USA, Canada
ALLHAT_LIPID 2008	10060	4.8	66.6	51.2	HTN, CVD, T2DM, CKD	–	78.5±19	–	–	35.1	100	36.6	USA, Canada
TRANSCEND 2009	5926	4.6	66.9	57	CVD, T2DM	1.0±0.3	–	10.3	0	35.7	76.4	74.5	Multicontinental
ONTARGET 2008	25620	4.6	66.4	73	CVD, T2DM	1.1±0.2	–	13.2	–	38	69	85	Multicontinental
HOPE 2000	3577	4.5	65.4	63	CVD, T2DM	1.0±0.3	–	32	0	98	56	60	Europe, Canada
BENEDICT-B 2011	281	4.5	62.3	73.7	HTN, T2DM	0.9±0.2	–	100	0	100	100	–	Multicontinental
ADVANCE 2007	11140	4.3	66	57	T2DM, CVD	0.9±0.3		26	4	100		32	Multicontinental
DIRECT 2009	3322	4	40.3	54.6	T1DM	1.0±0.2	–	0	0	100	23	–	Multicontinental
DIABHYCAR 2004	4912	4	65.1	69.8	T2DM	1.0±0.2	–	74	26	100	55.7	24.4	Multicontinental
Fogari R et al 2002	309	4	62	56	T2DM, HTN	1.0±0.5	89.6±14.1	100	0	100	100	0	Italy
Facchini F et al 2003	191	3.9	59.5	50.5	T2DM, CKD	1.8±0.6	63±30	0	100	100			USA
Steno Type 2 1999	160	3.8	55.1	74	T2DM	0.8±0.1	117±24.5	100	0	100		20	Denmark
BENEDICT 2004	1204	3.6	62	52.7	HTN, T2DM	0.9±0.2	–	0	0	100	100	–	Multicontinental
ACCORD 2010	10251	3.5	62		T2DM, CVD	0.9 (0.7–1.0)	90 (76–105)	26.5	6.5	100			USA, Canada
RENAAL 2001	1513	3.4	60	63	T2DM, HTN, CKD	1.9±0.5	–	0	100	100	96	11	Multicontinental
ROADMAP 2011	4447	3.2	57.7	46.1	T2DM	0.8±0.2	84.9±17.2	0	0	100		24.8	Multicontinental
CASE-J 2008	4703	3.2	63.8	55.1	HTN, T2DM, CVD, CKD	–	–	–	–	43	100	43.2	Japan
AIPRI 1996	583	3	51	72	CKD	2.1±0.6	42.6±11.1				82		Multicontinental
CSG 1993	409	3	34.5	53	IDDM, HTN	1.3±0.4	81.5±40.5			100	75.5		USA
AASK 2001	1094	3	54.3	61	CKD with HtnNS, CVD	1.9±0.7	46.4±13.4	–	33	0	100	52.5	USA
Hannedouche T et al 1994	100	3	51	53	CKD, HTN	3.0±0.1	–	0	100	0	100	0	France
ACCOMPLISH 2010	11506	2.9	70	64	CVD, HTN, DM, CKD	1.3±0.3	63.8±14	35	19	59.7	100	100	Multicontinental
ACTION I 2004	690	2.8	39	59.3	T1DM, CKD	1.6±0.4	60±29	0	100	100		83	USA, Canada
DIVINe 2010	252	2.7	60.4	74.5	DM, CKD, HTN	1.5±1.0	54±27	0	100	82	93.7	31	Canada
Ruggenenti 1999	186	2.7	49.7	75	HTN, CKD	2±0.1	46.5±18.2	0	100	0	82	–	Italy
IDNT 2001	1148	2.6	58.9	66.3	T2DM, HTN, CKD	1.6±0.5	–	0	100	100	100	28.6	Multicontinental
ATLANTIS 2000	134	2	40	45	IDDM, HTN	–	104±26	100	0	100	–	–	UK, Ireland
Laffel L, et al 1995	143	2	32.7	50.3	T1DM	1.1±0.2	80±21.5	100	0	100	0	0	USA, Canada
MCSG 1996	235	2	32	52	IDDM	0.9±0.2	95±35.5	100	0	100	0	0	Multicontinental
IRMA 2 2001	590	2	58	68	T2DM, HTN, CVD	1.0±0.1	109±2	100	0	100	100	26.6	Multicontinental
Stefoni S et al 1996	189	2	53.2	67	CKD, HTN	2.3±0.1	40.8±1.5	0	100	0			Italy
SURE 2009	205	2	66	66.5	T2DM, HTN, CKD					100	96	15	China
Val-HeFT 2009	5010	1.9	62	77	CVD, CHF, CKD, DM	–	59.8±10.5	–	–	33.7	7.5	45	USA
Goicoechea M et al 2010	113	1.9	71.8		CKD	1.8±0.5	40±11.9			37.5		27.5	Spain
REIN 2 2005	335	1.5	53.7	75	CKD	2.7±1.1	35±18.3	0	100	0			Italy
REIN 1997	166	1.3	49.3	78	HTN, CKD	2.4±0.9	38.8±18.2	0	100	0	87		Italy
BEAM 2011	227	1	66.8	56	T2DM, CKD	2.0±0.5	32.4±6.9	29	34	100			USA
CAP-KD 2009	479	1	63	33	CKD	2.6±1.0	22.3±10.8						Japan
DIAL 2004	180	1	59	73	T2DM, HTN	0.8±0.2	–	100	0	100	100	4	Italy
GUARD 2008	304	1	57.7	65.4	T2DM, HTN	–	90.6 (46.2–177.5)			100	100		USA
NESTOR 2004	570	1	60	64	T2DM, HTN	–	92.5±29.3	100	0	100	100		Multicontinental

We pooled studies by type of intervention and assessed the effects on renal endpoints (Table 2). Of all the interventions, only RAAS blockade (ACEi or ARB) vs. placebo consistently reduced renal endpoints as evidenced by a pooled upper bound of the 95% confidence interval (CI) <1 with at least 3 individual studies having an upper bound of the 95% CI <1. Thus, RAAS blockade was deemed “efficacious for prevention of incident or progressive CKD” and advanced to the second phase of our analysis. There were 18 trials of RAAS blockade vs. placebo [17], [18], [21], [24], [29], [31], [40], [47]–[51], [53], [54], [57], [69], [70]. Two of these trials had two treatment arms which were compared with the placebo separately. [54], [57] We report these data as two separate comparisons vs. placebo within each of these two trials, thus the total comparisons would be equal to 20 in the subsequent analyses.

**Table 2:** Studies included in Phase I Analysis grouped by Intervention.

ACEi and/or ARB vs Placebo (N = 32557)	ACEi vs ACEi plus ARB
• Hope 2000	• ONTARGET 2008
• DIABHYCAR 2004	**ACEi vs Beta Blocker (N = 100)**
• AIPRI 1996	• Hannedouche T, et al 1994
• CSG 1993	**ACEi vs CCB (N = 1274)**
• Ruggenenti 1999	• AASK 2001
• ATLANTIS 2000	• DIAL 2004
• Laffel L, et al 1995	**ACEi vs Diuretic (N = 33927)**
• MCSG 1996	• NESTOR 2004
• REIN 1997	• ALLHAT_BP 2005
• TRANSCEND 2009	**Allopurinol vs Control (N = 113)**
• DIRECT 2009	• Goicoechea M, et al 2010
• RENAAL 2001	**Bardoxolone vs Placebo (N = 227)**
• ROADMAP 2011	• BEAM 2011
• IRMA2 2001	**CCB vs Placebo (N = 2352)**
• Val-HeFT 2009	• BENEDICT 2004
• Mauer M, et al 2009	**CCB vs Diuretic (N = 33357)**
• BENEDICT 2004	• ALLHAT_BP 2005
• IDNT 2001	
**Intensive vs Conventional Glycemic Control (N = 29353)¥**	**Conventional therapy vs Conventional plus AST-120 therapy for CKD (N = 479)**
• UKPDS34 1998	• CAP-KD 2009
• UKPDS33 1998	**CCB vs ARB (N = 5851)**
• DCCT 1993	• CASE-J 2008
• Kumamoto 1995	• IDNT 2001
• VADT 2009	**Intensive vs Conventional BP Control (N = 335)**
• ADVANCE 2008	• REIN 2 2005
• ACCORD 2010	**Ibopamine vs Control (N = 189)**
**Usual vs Structured Care of CKD (N = 506)** *	• Stefoni S, et al 1996
• Rachmani R, et al 2004	**Statin vs Placebo (N = 18896)** *
• Steno Type 2 1999	• 4S
• SURE 2009	• AFCAPS-TexCAPS 2010
**ACEi plus CCB vs ACEi (N = 509)**	• ALLHAT_LIPID 2008
• BENEDICT-B 2011	**Statin plus Ezetimibe vs Placebo (N = 9270)**
• Fogari R, et al 2002	• SHARP 2011
**ACEi plus CCB vs Placebo (N = 1204)**	**Pimagedline (AGE Inhibitor) vs Placebo (N = 690)**
• BENEDICT 2004	• ACTION I 2004
**ACEi plus CCB vs ACEi plus Diuretic (N = 11810)**	**CR-LIPE Diet vs Control (N = 191)**
• ACCOMPLISH 2010	• Facchini F, et al 2003
• GUARD 2008	**Vitamin B vs Placebo (N = 252)**
**ACEi plus CCB vs CCB (N = 309)**	• DIVINe 2010
• Fogari R, et al 2002	
**ACEi plus Diuretics vs Placebo (N = 11140)**	
• ADVANCE 2007	

¥ Micro and macroalbuminuria were the only renal outcomes improved by intervention. No beneficial effect of intervention reported for other renal outcomes (doubling of creatinine, ESRD), thus not forwarded to phase II analysis.

* No beneficial effects seen for renal outcomes by this intervention thus not forwarded to phase II analysis.

ACEi Angiotensin converting enzyme inhibitor; ARB Angiotensin receptor blocker; CCB Calcium channel blocker; AST-120 an oral adsorbent agent; AGE Advanced glycation endproduct; CR-LIPE carbohydrate restricted low iron available polyphenol enriched diet.

A total of 32,557 patients were enrolled in these 18 RAAS trials. Study populations include adults with a mean age of participants 50.8 years. Approximately 62% were male. The median follow up time in these trials was 3 years with a range of 1.3 to 4.6 years. About 83% trials were in the setting of diabetes mellitus and 6 trials (33%) included patients with CKD (stage III or more and/or macroalbuminuria) [18], [22], [23], [25], [30], [31], [35], [39], [41], [43], [46], [53], [55], [60], [66], [67], [70]. The overall quality of studies included was good. All but 4 trials had low risk of selection bias due to random sequence generation although majority did not mention any specific methods for allocation concealment. Performance and detection biases were also low. About 25% trials had high risk of reporting bias due to selective reporting (Figures S1A and B).

### Renal Endpoints

**Figure 2:**
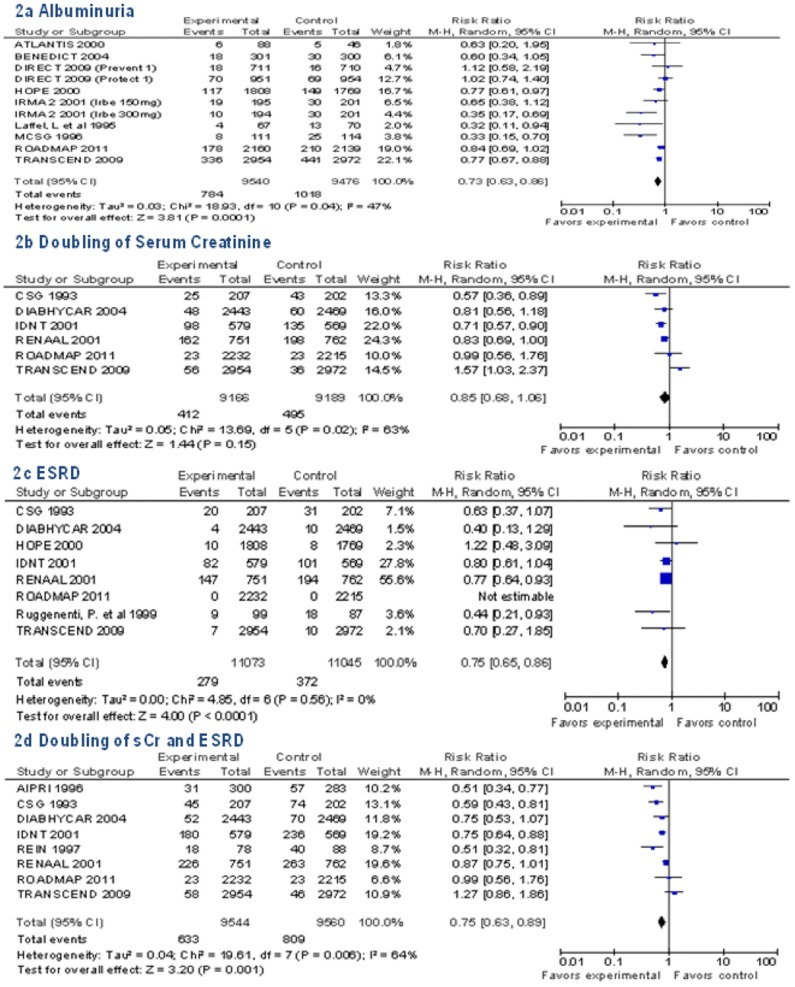
Pooled Relative Risk for Renal Endpoints in Renin-Angiotensin-Aldosterone-System (RAAS) Trials.

RAAS blockade vs. placebo was effective for the following renal endpoints: incident albuminuria was reduced by 27% (n = 11 trials, pooled RR 0.73 [95% CI 0.63 to 0.86], risk difference (RD) −3 [95% CI −5 to −1], I^2^ = 47%); ESRD by 25% (n = 8 trials, RR 0.75, [95% CI 0.65 to 0.86], RD −1 [95% CI −2 to 0], I^2^ = 0%); the composite endpoint of doubling of sCr and ESRD by 25% (n = 8 trials, RR 0.75 [95% CI 0.63 to 0.89], RD −4 [95% CI −6 to −2], I^2^ = 94%). There was a non-significant trend toward benefit for the endpoint of doubling of sCr (n = 6 trials, RR 0.85 [95% CI 0.68 to 1.06], RD −1 [95% CI −3 to 0], I^2^ = 90%), as shown in Figure 2.

### Mortality

**Figure 3:**
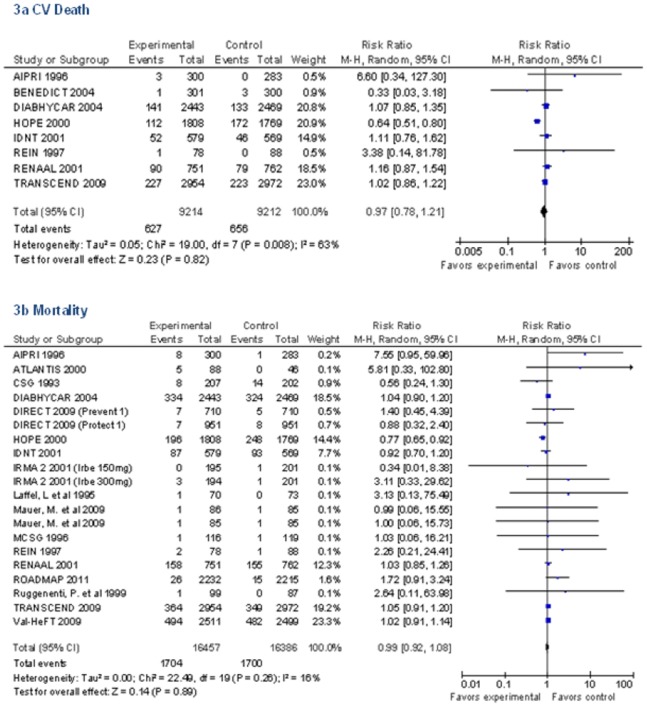
Pooled Relative Risk for Mortality in RAAS Trials.

Only eight RAAS trials reported on the outcome CV death (Figure 3a and Table 3). There were 627 deaths (6.8%) in treatment group vs. 656 deaths (7.1%) in control group (RR 0.97 [95% CI 0.78 to 1.21], RD 0 [95% CI −1 to 1], I^2^ = 67%). All 18 RAAS trials (20 total comparisons due to multiple arms in two trials) reported all-cause mortality (Figure 3b and Table 3). There were 1704 deaths (10.4%) in the RAAS treatment group vs. 1700 deaths (10.4%) in the control group (RR 0.99 [95% CI 0.92 to 1.08], RD 0 [95% CI 0 to 1], I^2^ = 29%). We had at least 80% power to detect a 14% relative risk reduction (RRR) in CV death and a 9% RRR in all-cause mortality at 2 sided alpha of 0.05 assuming baseline event rate in controls to be 7.1% and 10.4% respectively (Table 3).

**Table 3:** Incidence and power calculations for effect size in RAAS Trials.

Outcome	No. of studies	Sample Size	Incidence in Control Group n (%)	Incidence in Treatment Group n (%)	RR (95% CI)	Required RR for 80% power	Power With Current Event Rate
CV Death	8	18426	656 (7.1)	627 (6.8)	0.97 (0.78, 1.21)	0.86	8%
All-cause Mortality	20	32843	1700 (10.4)	1704 (10.4)	0.99 (0.92, 1.08)	0.91	5%

Abbreviations: RAAS =  Renin angiotensin aldosterone system; CV =  cardiovascular; RR =  relative risk.

### Sensitivity Analyses

We examined mortality only in the positive trials that demonstrated a statistically significant benefit for the renal endpoints. 8 trials reported on progressive renal disease as doubling of sCr and ESRD. 6 out of these 8 trials were protective for progressive renal disease (RR 0.70 [95% CI 0.59 to 0.82], RD −10 [95% CI −18 to −1], I^2^ = 94%), yet, in these 6 positive trials for CKD, there was no benefit in terms of all-cause mortality (RR 1.01 [95% CI 0.87 to 1.17], RD 1 [95% CI −1 to 2], I^2^ = 32%) or CV death (n = 5 trials, RR 1.11 [95% CI 0.95 to 1.31], RD 1 [95% CI 0 to 2], I^2^ = 0%). 11 trials reported on albuminuria out of which 6 were protective for albuminuria (RR 0.68 [95% CI 0.55 to 0.84], RD −4 [95% CI −7 to −2], I^2^ = 74%), however in those 6 positive trials, there was no benefit for all-cause mortality (RR 1.02 [95% CI 0.77 to 1.35], RD 0 [95% CI −1 to 1], I^2^ = 74%). Only two of these 6 trials reported CV death.

In order to further explore the causal relationship between the CKD and mortality within the group of RAAS vs. placebo trials, we performed several sub-group analyses and meta-regression by study-level variables and their association with the RR for mortality. When study-level variables were examined categorically, we found a non-significant trend towards reduction in mortality via RAAS blockade by length of follow up time, such that there was absolutely no benefit seen for studies with follow-up time <2 years and 2 to 4 years, but there was a suggestion of protection with follow-up time >4 years (RR 0.91 [95% CI 0.71 to 1.17], RD −1 [95% CI −3 to 1], I^2^ = 61%). However, only 4 trials had median follow-up greater than 4 years, and the statistical heterogeneity for this subgroup was high. There was no substantial differences in the pooled estimates when stratified by sample size (<1500 vs. >1500 participants), by mean GFR of study participants (>60 vs. ≤60 ml/min/1.73****m^2^), by studies that enrolled participants exclusively with diabetes vs. other trials, by the proportion of participants in each trial with albuminuria (Table 4). Meta-regression was used to explore the association between three continuous study level variables and the risk of death: A) ARR of ESRD and doubling of sCr between RAAS blockade vs. placebo; B) ARR of albuminuria by RAAS blockade vs. placebo; C) and overall study sample size. None of the three study level characteristics explained a significant proportion of variability in the risk of death. ARR of albuminuria explained 13% of the variability, sample size explained 11%, and ARR of ESRD and doubling of sCr explained 3% of the variability (Figure S2).

**Table 4:** Sub analyses of pooled relative risk of mortality.

Stratification	Number		Pooled RR	I^2^
	Studies	Participants	(95% CI)	
**Follow up**				
***≤2 years***	7	6,278	1.03 (0.92–1.15)	0%
***>2–4 years***	9	16,520	1.03 (0.90–1.18)	34%
***>4 years***	4	9,759	0.91 (0.71–1.17)	58%
**Sample Size**				
***<1500***	10	3,816	0.93 (0.73–1.19)	0%
***>1500***	8	28,707	1.00 (0.90–1.10)	44%
**eGFR**				
***>60 ml/min/1.73*** **** ***m^2^***	14	25,251	0.97 (0.86–1.08)	19%
***≤60 ml/min/1.73*** **** ***m^2^***	4	7,272	1.04 (0.89–1.21)	25%
**Diabetes**				
***No***	4	11,685	1.04 (0.92–1.18)	26%
***Yes***	14	20,838	0.96 (0.85–1.07)	13%
**Albuminuria**				
***None***	5	8,110	1.39 (0.87–2.22)	0%
***up to 33%***	2	9,503	0.91 (0.67–1.22)	86%
***>33%***	8	8,908	1.02 (0.92–1.14)	0%
***Microalbuminuria***	7	15,584	0.96 (0.82–1.12)	43%
***Macroalbuminuria***	3	2,827	1.00 (0.85–1.17)	0%

Abbreviations: RR =  relative risk; I^2^ =  heterogeneity; eGFR =  estimated glomerular filtration rate.

## Discussion

In this systematic review and meta-analysis, we sought to examine if the risk reduction in renal endpoints in randomized clinical trials also leads to a decrease in mortality. Despite the fact that RAAS blockade was beneficial for reduction in several renal endpoints (albuminuria, doubling of sCr and ESRD), we could not obtain firm evidence that renal benefit also translated into improved mortality, either all-cause or CV death.

These results are contrary to those observed in observational studies. A large systematic review and meta-analysis of 41 studies showed that point estimate for the unadjusted relative risk of mortality in patients with CKD (vs. those without CKD) exceeded 1.0 in 40 studies and was significant in 93% cohorts. The overall pooled relative risk for mortality for CKD vs. no CKD was 1.77 (95% CI 1.33 to 2.34). [10] Meta-regression analyses revealed an increasing risk of mortality with decreasing renal function. These findings, though observational, would imply that mortality can be reduced when there is a reduction in CKD outcomes. However, despite examining the outcomes in RCTs containing over 32,000 participants in trials of RAAS blockade vs. placebo, we could not confirm the implications from the observed associations between CKD and mortality.

We had enough statistical power to detect differences in mortality and CV death of 9 to 14%. However, the point estimates for all-cause mortality and CV death were very close to 1, making it unlikely that larger sample size resulting in more statistical power would have demonstrated a meaningful reduction in mortality. Thus, our findings leave a few possibilities that warrant careful consideration: i) CKD is causal for mortality and CV mortality but the duration of follow-up of the trials was not sufficiently long enough to witness the reduction in these outcome, ii) the effect size on CKD by RAAS blockade was too small to translate into a reduction in mortality, iii) the etiologic fraction of CKD for mortality and CV mortality is too small and requires larger sample size, iv) the event rate of mortality and CV mortality was too low; v) CKD is associated with mortality although a direct causal pathway between them is not existent.

Was the length of follow-up in the trials not sufficiently long enough to witness a beneficial effect on mortality? The median follow up time in the trials included in our meta-analysis was 3 years for the RAAS trials. When we examined mortality by doing the sub-analysis of studies stratified by the median duration of follow-up, we observed a non-significant trend towards reduced mortality in the 4 studies with median follow-up >4 years. Recently, the data from two large CKD intervention trials with prolonged follow-up (22 years in The Diabetes Control and Complications Trial/Epidemiology of Diabetes Intervention and Complications [DCCT/EDIC study] and 12 years in African American Study of Kidney Disease [AASK study]) were published [71], [72]. The AASK collaborative group reported a 24% reduction in doubling of sCr and ESRD in patients with urine protein to creatinine ratio (PCR) of >0.22 at 12 years but there was no improvement for mortality (Hazard Ratio 0.98, 95% CI 0.65 to 1.46). Data from DCCT/EDIC study showed that intensive glycemic control resulted in a 46% reduction in the endpoint of impaired eGFR, but there was no significant difference in mortality (HR 0.88, 95% CI 0.54 to 1.42). These two studies, despite very long follow up time, could not demonstrate that improvement in CKD outcomes manifested in a reduction in mortality, although by themselves, they were underpowered to detect a difference in mortality.

Was the effect size afforded by RAAS blockade for the renal outcomes too small to translate into reduction of mortality? In the RCTs herein, the relative risk reduction for renal outcomes was 15 to 27%, and the absolute risk reduction only ranged from 1 to 4%. Even if the mortality benefit completely paralleled that for CKD, then the largest absolute difference in mortality between the 2 interventions can only be 4%. However, the benefits of renal protection are not transmitted 100% towards the benefit of the hard outcome. This relates to the concept of etiologic fraction. The “etiologic fraction” or “attributable risk exposed” of CKD for non-renal outcomes lies somewhere between 0 to 99%. Using the data from meta-analysis by Tonelli et al. [10] the attributable proportion in the total population (Ap_t_) of CKD for death is 34.1%, (Table 5) which means a 4% reduction in the risk of CKD will translate into a 1.3% reduction in all-cause mortality.

**Table 5:** Calculation for Attributable Proportion of CKD for All-cause Mortality.

	Mortality	No Mortality	Total
**CKD**	***a*** 24420	***b*** 173104	197524
**No CKD**	***c*** 29437	***d*** 928376	957813
**Total**	53857	1101480	Grand Total (a+b+c+d) 1155337

AP_t_ = [(R_t_–R_u_)/R_t_]×100.

AP_t_ is Attributable Proportion of mortality due to CKD in the total population.

R_t_ is the prevalence of mortality in the total population = a+c/a+b+c+d.

R_u_ is the prevalence of mortality in the unexposed (without CKD) population = c/c+d.

R_t_ = 53857/1155337 = 4.66%.

R_u_ = 29437/957813 = 3.07%.

AP_t_ = 34.1%.

The 4^th^ possibility relates to the event rate of the hard outcomes in the trials. In order to appreciate a sizeable effect on an outcome, the event rate needs to be of a certain magnitude in order to influence it. The mortality rate in the RCTs ranged from 0.0 [47] to 21.65 [19]. Observational studies have demonstrated a mortality rate as high 6.8/100 person-years in patients with CKD [73]. Due to insufficient data to perform meta-regression in order to explore the association of event rate in studies with the risk of mortality, we are unable to conclude if a higher event rate would have explained the present lack of mortality benefit.

The last possibility is that CKD may not be directly causal for CV death and overall mortality. This notion although seems provocative and contrary to common belief in the nephrology community, to disregard it completely as a heresy may not be the right approach. Another way to look at this association would be to see the increased mortality in patients in which CKD is induced or incidence is increased iatrogenically. On the contrary, Lau, et al. have shown the renal cell carcinoma patients undergoing radical nephrectomy were at a 3-fold higher risk of developing CKD when compared to those who underwent nephron sparing surgery. Yet there was no difference in mortality in the two groups at 10 years [74]. Moreover, Wald, et al. used propensity-based methods to match patients with and without severe AKI. After a median follow up time of 3 years, they demonstrated that those who experienced AKI had a 3-fold increased risk of ESRD. Nevertheless, despite the fact that there were 3 times as many patients who developed ESRD compared to the no AKI group, there was absolutely no difference in all-cause mortality [12]. The discordant association between CKD risk and mortality, in both directions, provides some equipoise around the assumption of a direct causal pathway between CKD and mortality. We as clinicians and researchers have witnessed several times over the past few years that approaching the targets based on surrogate markers has not translated into clinical improvement. Recent, well designed trials to evaluate the effect of reduction of proteinuria did not show any significant improvement in hard outcomes [75], [76]. The same is true for anemia showing no improvement in clinical outcomes with correction of hemoglobin as well as HDL in patients with CVD [77]–[81]. The “independent associations” from observational studies may be confounded by other patient factors such as hypertension, dyslipidemia and diabetes that coexist with CKD. These established cardiovascular risk factors may be responsible for increased mortality witnessed in patients with CKD.

## Conclusions

We have definitive evidence to demonstrate a reduction in CKD endpoints by RAAS blockade. However, we still lack conclusive evidence that the reduction in CKD endpoints will translate into a meaningful reductions in CV and all-cause mortality. To date, evidence from RCTs is not sufficient to fulfill the most important condition to prove the causality between CKD and mortality. However, the results of our analysis cannot be generalized to other interventions that may reduce or prevent CKD. For example, at the present time, there is insufficient data published for some interventions, such as allopurinol and alkali therapy, in CKD patients and the effect on mortality [82]–[84]. We suggest that unless more potent and efficacious agents for prevention or treatment of CKD are discovered (to increase the effect size), then RCTs of RAAS blockade (or other similarly effective agents) will need to be performed with longer duration of follow-up time than typically performed in CKD trials (at least 4 years or more of follow-up) in order to prove that direct causal relationships between CKD and mortality exist. Until then, we are unclear whether CKD shares the same company as other surrogate and non-causal endpoints in nephrology, such as anemia and vitamin D deficiency. [78], [79], [81], [85].

## Supporting Information

Figure S1 Risk of Bias Summary for each included trial (1A). Risk of Bias Graph presented as percentage across all included trials (1B).
(TIF)

Figure S2 **Meta-regression Analysis of Association between Study Level Covariates and Risk of All-cause Mortality.**
(TIF)

Checklist S1 **PRISMA 2009 Checklist.**
(DOC)
