# Supplementary figures and images for: Prevention of Chronic Kidney Disease and Subsequent Effect on Mortality: A Systematic Review and Meta-Analysis
Source: PLoS One. 2013 Aug 29;8(8):e71784. doi: 10.1371/journal.pone.0071784 (PMC3756976; doi:10.1371/journal.pone.0071784)

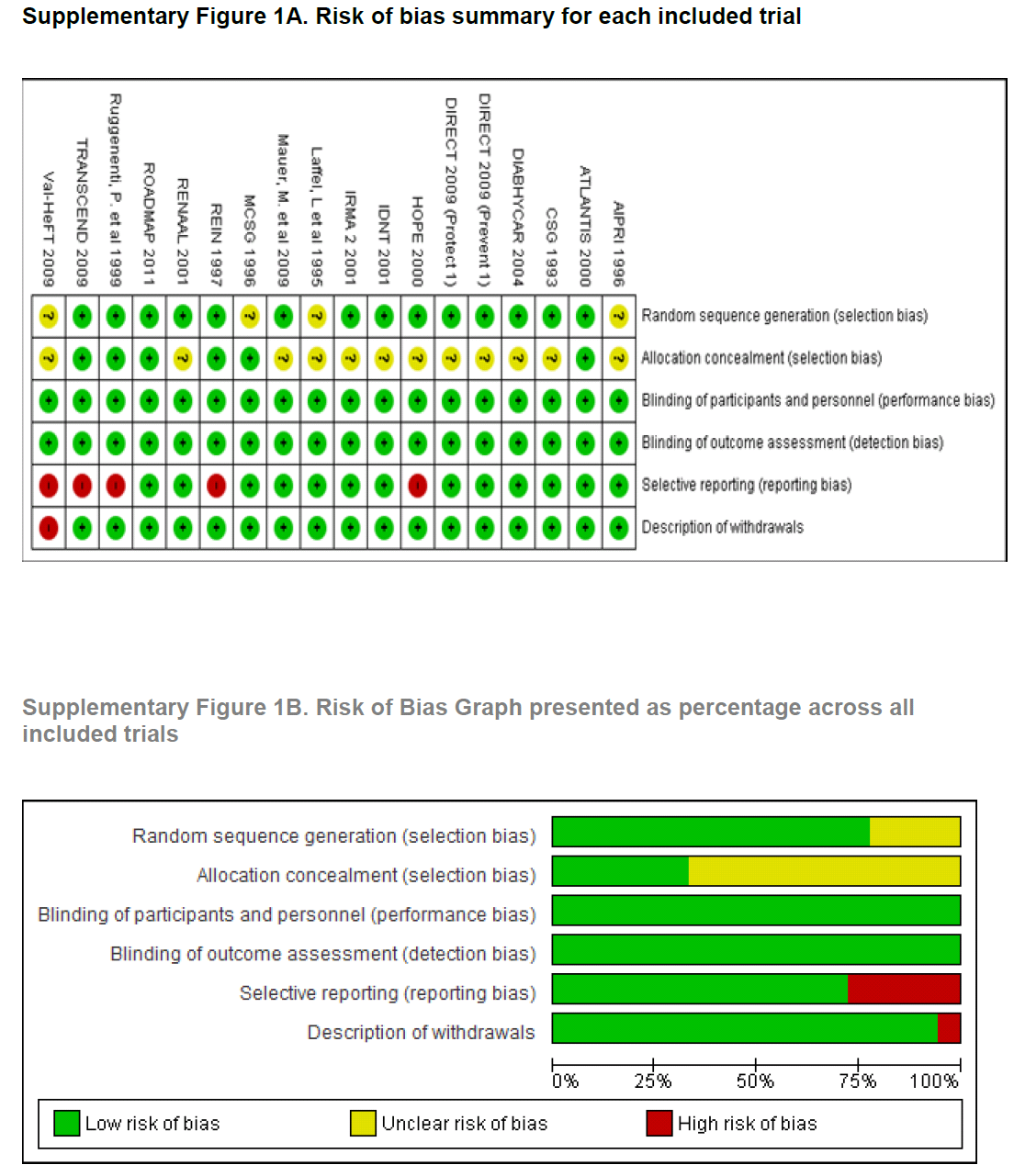

Supplement: Figure S1 — Risk of Bias Summary for each included trial (1A). Risk of Bias Graph presented as percentage across all included trials (1B). (TIF) [file pone.0071784.s001.tif]

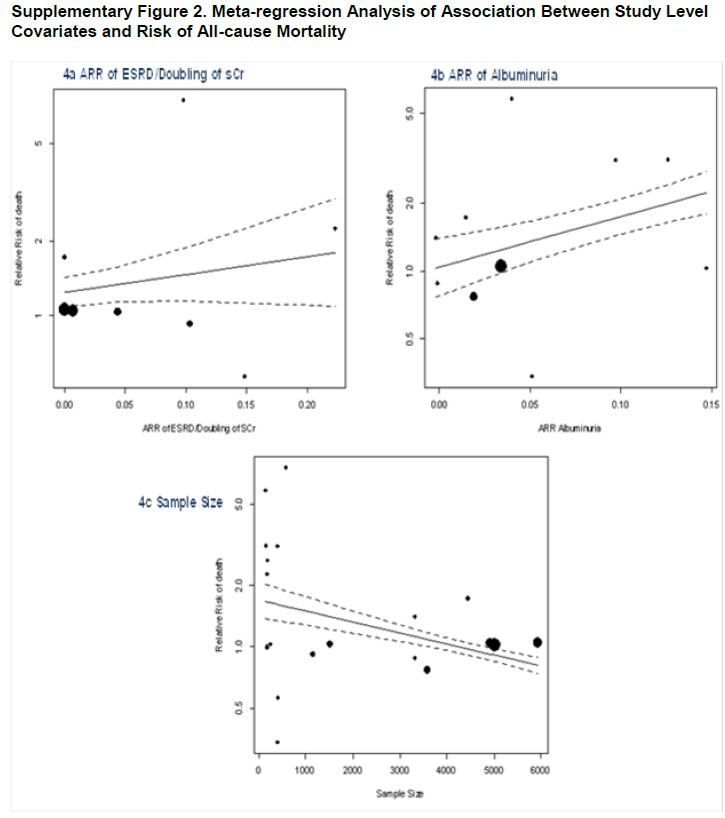

Supplement: Figure S2 — Meta-regression Analysis of Association between Study Level Covariates and Risk of All-cause Mortality. (TIF) [file pone.0071784.s002.tif]
